# The Functional Vision Restorative Effect of Crocin via the BDNF–TrkB Pathway: An In Vivo Study

**DOI:** 10.3390/nu14091716

**Published:** 2022-04-20

**Authors:** Jia-Lain Wu, Shih-Liang Yang, Yung-Chuan Ho, Chao-Hsiang Chen, Bing-Rong Tasi, Meng-Chih Lee, Bo-Yie Chen

**Affiliations:** 1 Department of Optometry, Chung Shan Medical University, Taichung 40201, Taiwan; gloriawu7@gmail.com (J.-L.W.); judywolfsam@gmail.com (B.-R.T.); 2 Department of Chinese Medicine, Taichung Hospital, Ministry of Health and Welfare, Taichung 40343, Taiwan; ysl451ysl@yahoo.com.tw; 3 Department of Medical Applied Chemistry, Chung Shan Medical University, Taichung City 40201, Taiwan; ych065@csmu.edu.tw; 4 Department of Ophthalmology, Chung Shan Medical University Hospital, Taichung 40201, Taiwan; 5 Graduate Institute of Pharmacognosy, Taipei Medical University, Taipei 11031, Taiwan; cmy@koda.com.tw; 6 Ko Da Pharmaceutical Co., Ltd., Taoyuan 32459, Taiwan; 7 Institute of Medicine, Chung Shan Medical University, Taichung 40201, Taiwan; mengchihlee@gmail.com; 8 Institute of Population Health Sciences, National Health Research Institutes, Miaoli 35053, Taiwan

**Keywords:** crocin, BDNF, TrkB, vision protection, photoreceptor, functional vision, visual contrast sensitivity function

## Abstract

Abnormal dislocation of cone opsin protein affects the sensitivity function of photoreceptors and results in depressed central vision. Nutraceutical therapy is needed to restore the residual function of photoreceptors. Crocin is a natural substance for retinal health. However, its effect on the restoration of functional vision and its underlying mechanisms have not been fully studied. This study analyzed the restorative effect of crocin on residual functional vision in vivo in a mouse model. High-energy light-evoked photoreceptor dysfunction was confirmed by M opsin dislocation in the retina accompanied by a loss of functional vision. Crocin treatment significantly increased brain-derived neurotrophic factor (BDNF) protein in retinas, thus contributing to the re-localization of the M opsin protein, restoration of the visual acuity (VA), and high spatial frequency-characterized visual contrast sensitivity function (VCSF). In contrast, such effects were significantly reversed after the washout period. Additionally, the restorative effect of crocin on functional vision and M opsin re-localization can be reversed and blocked by synchronous injection of a tropomyosin receptor kinase B (TrkB) receptor antagonist (ANA-12). This study demonstrated the major functional vision-rescuing or restoring effect of crocin in vivo by modulating M opsin location plasticity and increasing the capacity of the residual photoreceptor function through the BDNF–TrkB receptor pathway.

## 1. Introduction

Light-evoked maculopathy, age-related macular degeneration (AMD), and diabetic maculopathy affect a person’s central vision [1,2,3,4,5]. Functional vision abnormalities, especially loss of visual acuity (VA) and high spatial frequency-characterized visual contrast sensitivity function (VCSF), may accompany or precede clinically detectable morphological damage in the retina [6,7,8]. Additionally, the impact of blue light hazard or high-energy visible light has been demonstrated to contribute to photoreceptor impairment and dysfunction in vivo [9,10,11]. Persistent excessive exposure to artificial high-energy visible light might alter the functions of central vision or aggravate the progression of early or late maculopathy [5,12]. Light-evoked photoreceptor changes initiate functional vision abnormalities. Although there is no cure for maculopathy, there are nutrient treatment options that may prevent or slow the progression of the disease; however, it is also expected to restore residual photoreceptor function to enhance functional vision.

Crocin is considered the next vision supplement [13,14]. In recent studies, crocin-rich saffron and gardenia jasminoides have been explored and suggested for use as therapeutic supplements to treat maculopathy, diabetic retinopathy, and degenerative optic nerve diseases [13,14,15,16,17]. Our experiments have particularly indicated that pretreatment with crocin or crocetin could significantly preserve high spatial frequency-characterized VA and VCSF against light-evoked retinal damage in mice [11]. In addition, experiments have indicated that crocin or crocetin could protect light-evoked photoreceptors [14,18,19], reduce retinal ischemic damage [20,21], inhibit the development of proliferative vitreoretinopathy [22], ameliorate retinal vein occlusion-induced edema [23], block N-methyl-D-aspartate-induced retinal damage [24], attenuate the oxidative damage of retinal ganglion cells [25,26,27], and promote ocular circulation [28]. Furthermore, crocin can also clinically decrease diabetic macular edema and improve the best-corrected visual acuity (BCVA) [16]. Crocin rich-saffron supplementation modestly improved visual function and retinal flicker sensitivity in AMD [15,17]; however, it did not affect the VA and focal electroretinogram (fERG) response of the central retina in adenosine triphosphate (ATP) Binding Cassette Subfamily A Member 4 (ABCA4)-related Stargardt macular dystrophy [29]. 

The efficacy and power of crocin for vision restoration might be based on different pathological phases. Although electrophysiological methods of electroretinogram (ERG) are used in clinical or animal studies for the functional examination of photoreceptors, they suffer from low signal specificity and insufficient spatial resolution [30]. However, they do not represent the integrity of vision processing and vision response. The effect and efficacy of crocin in treating the progressive deterioration of visual function or enhancing the residual photoreceptor function have not been elucidated. In this study, optomotor response (OMR)-based VA and VCSF methods were used to investigate the efficacy of crocin and its underlying mechanism, involving residual functional vision enhancement or restoration in a mouse model of light-evoked damage. The brain-derived neurotrophic factor (BDNF)–tropomyosin receptor kinase B (TrkB) receptor pathway is important for photoreceptor survival against phototoxicity [31] and for cone photoreceptor visual function [32]. Colocalization of BDNF and TrkB proteins in green-red-sensitive cone outer segments has been indicated and might suggest they have specific role in the function of cones [33]. In this study, we investigated the role of the BDNF–TrkB pathway in the functional vision restoration effect of crocin. 

## 2. Materials and Methods

### 2.1. Animals

The animal model and experimental protocols were reviewed, and the sacrifice procedures were approved by the Institutional Animal Care and Use Committee (IACUC1937) of Chung Shan Medical University in accordance with the Guide for the Care and Use of Laboratory Animals. Approximately 8 to 12-week-old female CD1 (Institute of Cancer Research (ICR)) albino mice (*n* = 60), weighing 25–28 g, were purchased from BioLASCO Taiwan Co., Ltd. (Taipei, Taiwan). The mice had free access to food and water and were housed in a standard temperature- (23 ± 2 °C) and humidity- (55 ± 7%) controlled room.

### 2.2. Materials

Crocin (sc-217957, Santa Cruz, CA, USA) was freshly prepared in a vehicle containing 10% (v/v) propylene glycol 400 in distilled water and administered orally at 5 mg/kg twice a day (BID) and 50 mg/kg BID. The TrkB inhibitor, TrkB receptor antagonist (ANA-12) (SML0209, Sigma Aldrich, St. Louis, MO, USA), was freshly prepared in the same vehicle and administered intraperitoneally at 0.5 mg/kg, BID.

### 2.3. Experimental Design and Animal Groupings

**Figure 1:**
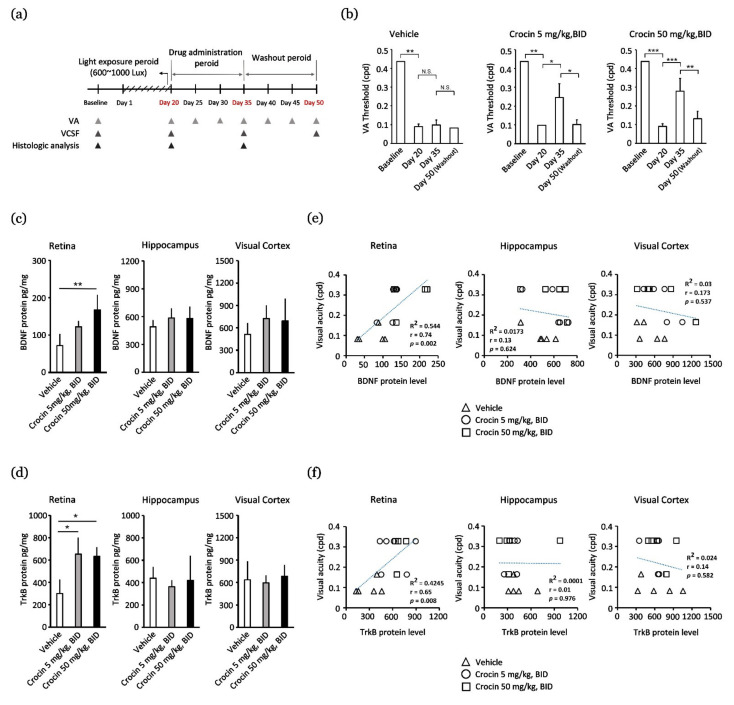
Crocin promotes visual acuity (VA) restoration via upregulating BDNF and TrkB protein content in retinas. (**a**) Timeline of the experimental design (the arrowheads indicate the occurrence at a given time); (**b**) recovery of VA by crocin treatment; (**c**) the BDNF protein content; (**d**) the TrkB protein content; (**e**) the correlation coefficient analysis of BDNF protein content and VA threshold; (**f**) the correlation coefficient analysis of TrkB protein content and VA threshold. Data are expressed as the mean ± standard error (SE). Mann–Whitney U test. *** *p* < 0.001, ** *p* < 0.01, and * *p* < 0.05. BDNF, brain-derived neurotrophic factor; TrkB, tropomyosin receptor kinase B; VCSF, visual contrast sensitivity function; N.S., non-significant; BID, twice a day; cpd, cycles per degree.

The light-evoked retinal damage model in mice was established based on our previous studies [10,11]; LED light exposure was performed (Figure 1a) (light intensity: 600–1000 lux with a 12 h:12 h light-dark cycle). To explore whether crocin can restore or improve residual functional vision after photodamaging retinal tissues as well as to examine its efficacy, a mouse model of continuous 20-day LED bright light-evoked exposure was generated. After LED bright light-evoked retinal photodamage was induced, oral treatment with (1) vehicle (*n* = 10), (2) crocin (5 mg/kg, BID) (*n* = 10), (3) crocin (50 mg/kg, BID) (*n* = 10), (4) combination treatment of vehicle + ANA-12 (0.5 mg/kg, BID., intraperitoneal injection (i.p.)) (*n* = 10), and (5) combination treatment of crocin (50 mg/kg, BID) + ANA-12 (0.5 mg/kg, BID, i.p.) (*n* = 10) was performed for 15 days, followed by a 15-day washout analysis period (Figure 1a). The timelines for the VA and VCSF assessments are indicated by arrowheads (Figure 1a); all mice were kept under dim-light conditions (50 ± 10 lux of illuminance) for 30 min before assessing the VA and VCSF [10,11]. VA and VCSF analyses were represented at day 0 (baseline, *n* = 10 per group), day 20 (*n* = 10 per group), day 35 (*n* = 10 per group), and day 50 (*n* = 5 per group). For retinal histological and protein analyses, mice were sacrificed on day 35 (*n* = 5 per group) and on day 50 (*n* = 5 per group). In addition, normal mice without LED light exposure were sacrificed at day 0 (baseline) as a normal blank group (*n* = 6), and at day 20 after LED bright light stimulation as an experimental control group (*n* = 4).

### 2.4. Determination of Thresholds of the VA and VCSF

Based on the methods used in our previous research, the VA and VCSF analyses administered to the mice were based on the optomotor response/reflex (OMR), whose thresholds were recorded by reflex responses that elicited compensatory head movements when their eyes were exposed to moving striped grating patterns [10,11] The VA and VCSF threshold levels of the mice were determined based on OMR recordings, until their response were no longer coordinated with the gratings. In the VA test, the patterns of grating stripes were set at 100% contrast and performed using six series of spatial frequencies at 0.033, 0.055, 0.082, 0.164, 0.328, and 0.437 cycles per degree (cpd), with a rotational speed of 12°/s drifting horizontally [10,11]. In the VCSF test, the described six striped grating patterns were subsequently set at 10 different contrast levels (%) to determine the individual threshold, and then drawn as an inverted U-shaped curve [10,11]. The area of the inverted U-shaped VCSF curve represents a VCSF visibility index that shows the overall capacity and characterization of spatial-frequency-based vision performance. Thus, if the mice responded to relatively lower-contrast stimuli, a higher VCSF visibility index was calculated to show a greater capacity for visual performance [10,11].

### 2.5. Histological and Immunohistochemistry Analyses

One of the mouse eyeballs was surgically removed, perfused with a fixative solution (3% formaldehyde, 5% glacial acetic acid, and 44% ethanol), and embedded in paraffin using a typical procedure. A 5 μm thick tissue section was prepared in the sagittal plane for hematoxylin and eosin (H&E) and immunohistochemistry (IHC) analyses. The retinal microstructure was analyzed using an Olympus CX-22 microscope (Olympus Corp., Tokyo, Japan), a Motic Moticam 3 camera, and Image software (version 2.0; Motic, Xiamen, China). For IHC analysis, heat sodium citrate buffer (10 mM sodium citrate, 0.05% Tween 20, pH 6.0) was used for antigen retrieval prior to incubation with the antibody. The experimental approach for signal detection was based on an IHC kit (Super Sensitive™ Polymer-HRP IHC Detection System, BioGenex Laboratories, Inc., San Ramon, CA, USA). In addition, M of opsin (1/250, Cat. No. NB110-74730, Novus Biologicals, Littleton, CO, USA) and TrkB antibodies (1/150, Cat. No. PA5-86241; Thermo Fisher Scientific, Inc., Waltham, MA, USA) were used in this study. The retinal outer nuclear layer (ONL) thickness as well as the average values of M opsin-labeled cells in the superior and inferior retinas were analyzed. The percentage of M opsin mislocalization in the ONL profile was analyzed to determine the pathological condition and physiological function of the photoreceptors [10].

### 2.6. Protein Analysis

The tissues of the retina, hippocampus, or visual cortex were isolated and homogenized with a protein extraction kit using proteinase inhibitors (PRO-PREP™ Protein Extraction Solution, iNtRON Biotechnology, Seongnam, Korea) and stored at −20 °C until analysis. BDNF (DBNT00, R&D Systems Inc., Minneapolis, MIN, USA) and TrkB proteins (ab 203368, Abcam Inc., Hangzhou, China) were detected using an ELISA kit.

### 2.7. Statistical Analysis

Statistical analyses were performed using SPSS version 22 software (IBM Corp., Armonk, NY, USA) and expressed as mean ± standard error (SE). The differences between the experimental groups were analyzed using Mann–Whitney U and Kruskal–Wallis tests. Associations between VA or VCSF and BDNF or TrkB protein content in the retina, hippocampus, or visual cortex were analyzed using Pearson’s correlation test. The results were considered significant at *p* < 0.05, *p* < 0.01, and *p* < 0.001.

## 3. Results

### 3.1. Crocin Restores the Thresholds of the Residual VA in Light-Evoked Retinal Photodamage Model

Light-evoked deterioration of the VA was observed on day 20. As a result, crocin-treated mice showed a significant restoration of VA threshold on day 35, which was subsequently reversed after the washout period on day 50 (*p* < 0.05 in the 5 mg/kg BID group, and *p* < 0.001 in the 50 mg/kg BID group) (*p* < 0.05 in the 5 mg/kg BID group, and *p* < 0.001 in the 50 mg/kg BID group) (Figure 1b). Crocin (50 mg/kg, BID) treatment resulted in a better VA restoration effect. A change in the VA threshold was not detected in the vehicle group; the residual VA remained low (Figure 1b). Therefore, crocin may functionally elevate the performance of the residual photoreceptor and/or the secondary vision-based neural recognition system.

### 3.2. Crocin Promotes VA Restoration via Upregulated Expression of BDNF and TrkB Protein in Retinas

We assessed BDNF and TrkB protein levels in the whole retina, hippocampus, and visual cortex on day 35 (Figure 1c,d). Crocin (5 mg/kg, BID, and 50 mg/kg, BID) treatment resulted in increased expression of BDNF and TrkB proteins, particularly in retinas, as compared to the vehicle group (Figure 1c,d). There was no difference in BDNF and TrkB protein content in the experimental group in the hippocampus and visual cortex. Moreover, the residual VA thresholds were correlated with BDNF and TrkB protein content in the retinas after LED light exposure, but not in the hippocampus or visual cortex (Figure 1e,f). In the retinas, the VA threshold was significantly correlated with BDNF (Pearson’s correlation coefficient; *r* = 0.74, *p* = 0.002; Figure 1e) and TrkB protein content (Pearson’s correlation coefficient; *r* = 0.65, *p* = 0.008; Figure 1f). These results indicate that crocin contributes to elevating functional vision by targeting retinal tissue and that BDNF and TrkB proteins might participate in this regulation.

### 3.3. Crocin Restores and Elevates the Residual Threshold of VA and VCSF via Activating the BDNF–TrkB Pathway

**Figure 2:**
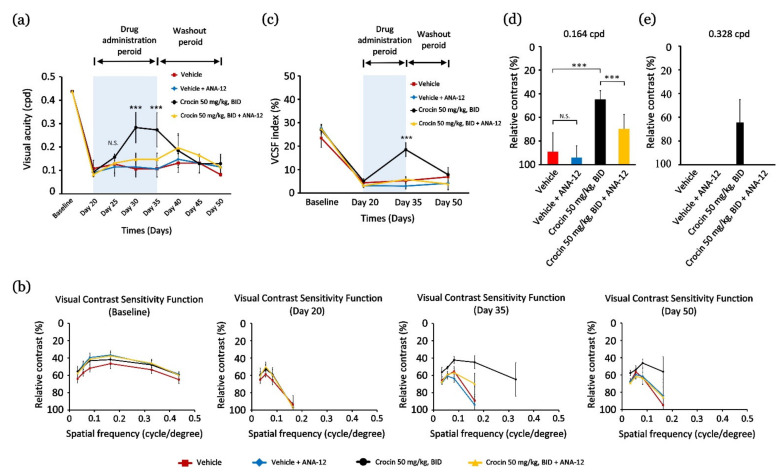
Crocin elevates and restores the thresholds of residual functional vision via activating the BDNF–TrkB pathway. (**a**) The change in the VA threshold following crocin treatment and washout; (**b**) the change in the VCSF threshold following crocin treatment and washout; (**c**) the change in the VCSF visibility index following crocin treatment and washout; (**d**) individual VCSF thresholds represented in 0.164 cpd; (**e**) individual VCSF thresholds represented in 0.328 cpd. Data are expressed as the mean ± SE. (**a**,**c**) Kruskal–Wallis test. (**d**) Mann–Whitney U test. *** *p* < 0.001. ANA-12, TrkB receptor antagonist.

Significant loss of VA (Figure 2a) and VCSF (Figure 2b,c) was observed after 20 days of LED light-induced retinal photodamage in mice. To investigate whether the BDNF–TrkB pathway is involved in the effects of crocin on functional vision promotion in vivo, a combination treatment of crocin (50 mg/kg, BID) and a BDNF–TrkB pathway antagonist (ANA-12; 0.5 mg/kg, BID, i.p.) was performed after 20 days of light exposure. As a result, on day 35, crocin (50 mg/kg, BID) treatment restored and elevated the VCSF threshold (Figure 2b) and VCSF index (*p* < 0.001, Figure 2c) as compared to the vehicle group, especially at middle-to-high-level spatial frequencies of 0.164 cpd (Figure 2b,d) and 0.328 cpd (Figure 2b,e). After the washout on day 50, the VCSF threshold and VCSF index were found to be significantly reversed, which demonstrated the direct effect of crocin (Figure 2b,c). Notably, after synchronous injection with ANA-12 in the crocin-treated group, the restorative efficacy of crocin on the VA threshold was significantly reversed on days 30 and 35 (*p* < 0.001, Figure 2a). In addition, the effects of crocin treatment on elevating the VCSF threshold (Figure 2b) and VCSF index (Figure 2c) were significantly reversed by synchronous ANA-12 injection. ANA-12 injection markedly reduced the recovery value of high spatial frequencies characterized by VCSF at 0.164 cpd (*p* < 0.001, Figure 2d) and 0.328 cpd (Figure 2e). Notably, upon injecting ANA-12 to crocin-treated mice, the VCSF threshold was not detected at 0.328 cpd (Figure 2e). In contrast, the injection dosage of ANA-12 (0.5 mg/kg, BID) in the vehicle-treated group did not influence the residual VA and VCSF thresholds. However, these results indicate that crocin may have activated the BDNF–TrkB pathway to restore or promote the residual function of retinal neurons to rescue VA and VCSF.

### 3.4. Crocin via BDNF–TrkB Pathway to Modulate M Opin Protein Localization to Function

**Figure 3:**
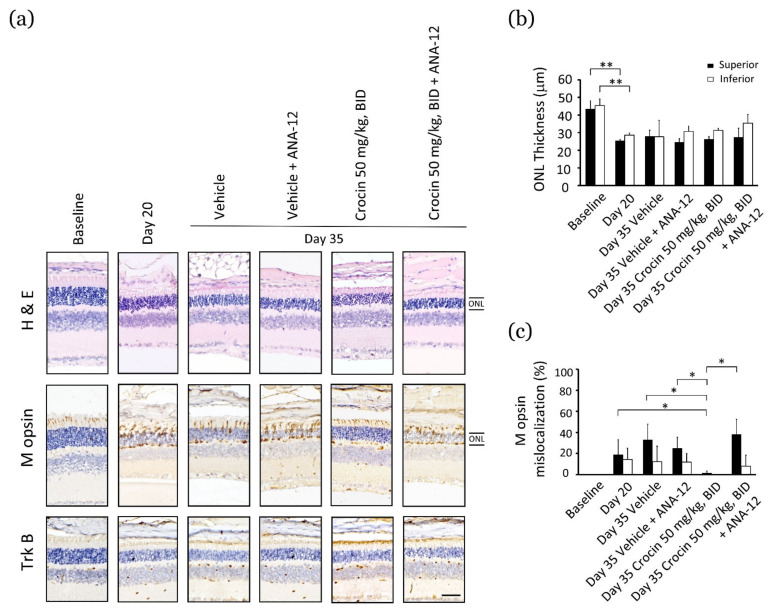
Crocin modulates M opsin protein localization to function via activating the BDNF–TrkB pathway. (**a**) The change in ONL thickness, M opsin protein, TrkB protein in retinas; (**b**) the average ONL thickness; (**c**) the percentage of M opsin mislocalization. Data are expressed as the mean ± standard error (SE). Mann–Whitney U test. ** *p* < 0.01, and * *p* < 0.05. Scale bar: 40 μm. ONL, outer nuclear layer; H&E, hematoxylin and eosin.

Histopathological microscopic examination indicated a significant decrease in ONL thickness in the retina after 20 days of high-energy light exposure (Figure 3a). There were no obvious changes in ONL thickness in the experimental group on day 35, as compared to the vehicle-treated group or the retina on day 20 (Figure 3b). These results indicate that the restorative effects of crocin-mediated vision might be independent of retinal tissue repair; however, it might functionally elevate the performance of the residual photoreceptors. As a result, photoreceptor function was impaired by mislocation of the M opsin protein in the ONL on day 20 compared to the normal retina (no light exposure) (Figure 3a,c). In particular, on day 35, crocin (50 mg/kg, BID) treatment significantly inhibited M opsin mislocation as compared to the vehicle-treated group or the retina on day 20 (Figure 3a, and *p* < 0.05, Figure 3c). Notably, synchronous injection of ANA-12 in the crocin-treated group significantly reversed the inhibitory effects of M opsin mislocation (Figure 3a, and *p* < 0.05, Figure 3c). In contrast, there was no obvious difference between the vehicle-treated and vehicle+ANA-12-treated groups (Figure 3a,c). Additionally, TrkB protein was predominantly increased in the outer segment of the photoreceptor in the crocin-treated group; expression was diminished by synchronous injection of ANA-12 (Figure 3a). These results indicate that crocin may activate the BDNF–TrkB pathway to modulate M opsin protein localization and to function in the outer segment of the photoreceptor, thereby enhancing the residual function of photoreceptors. 

## 4. Discussion

The healthy human macula consists of a small cone-dominated fovea that participates in photopic sensitivity and high spatial-frequency vision and is surrounded by a rod-dominated parafovea that participates in scotopic sensitivity and low spatial frequency vision [34,35]. Maculopathy is a fundamental characteristic of any disorder affecting the photoreceptors and is usually accompanied by deteriorating central vision; in patients, it is responsible for irreversible vision loss by progressive loss of high acuity and sensitive functional vision [34,36]. Recently, extracts of crocin-rich saffron and Gardenia jasminoides have been suggested as therapeutic supplements for retinal diseases [13,14,15,16,17,29]. In animal models, crocin and its hydrolyzed form, crocetin, possess retinal protective pharmacological properties [18,20,21,24,26]. Nevertheless, the therapeutic efficacy of functional vision restoration in vivo remains unclear. Therefore, this study was designed to evaluate the restorative effects of vision and the underlying mechanism of crocin on LED light-evoked retinal damage in vivo.

In this study of light-evoked damaged retinas, oral crocin treatment was demonstrated to functionally restore and elevate VA and VCSF thresholds by directly targeting residual photoreceptors as well as by activating the BDNF–TrkB pathway. In contrast, crocin-mediated activation of the BDNF–TrkB pathway in retinas contributed to the functional localization of the M opsin protein in the outer segment of photoreceptors. The dysfunctional photoreceptor with M opsin dislocation was reversed and reactivated during crocin treatment; however, the crocin-treated mice presented a higher response value for VA and VCSF than vehicle-treated mice on day 35. Notably, these values were significantly higher than the background of the 20-day light-evoked model, while the M opsin protein returned to the correct functional localization after crocin treatment. Additionally, the increased crocin-induced capacities of the residual photoreceptor function, M opsin location plasticity, and functional vision were reversed by synchronous ANA-12 injection. However, we also found that the upregulation of retinal BDNF and TrkB content was dramatically coordinated in crocin-treated cells. In this model of light-evoked degenerated retina background, even though crocin (50 mg/kg, BID) cannot directly contribute to ONL regeneration in retinas with light-evoked tissue damage, we demonstrated that crocin can significantly restore functional vision, especially in high spatial frequency VA and VCSF.

To date, only a few medical or nutritional therapies have been used to retard the progression of these diseases; there are no available therapeutic strategies for reversing tissue degeneration in the retina [36,37]. Consequently, the future treatment of maculopathy requires the development of formulas with natural active ingredients to enhance the residual photoreceptor function, in addition to antioxidative and anti-inflammatory capacities. The 20-day light-evoked retinal damage model in this study mimics the clinical conditions of early pathologic phases, thereby resulting in an easier recovery of retinal function. However, the capacity of crocin to directly enhance residual photoreceptor function and restore functional vision has been confirmed in this study; based on our results, the functional vision dramatically decreased during the washout period when the oral crocin treatment was stopped. Of interest, in our previous study, we demonstrated that crocin (0.25 mg/kg, BID and 5 mg/kg, BID) exhibits functional vision protection and enhanced activity [11]; here, we found that in order for it to function (50 mg/kg, BID), modulating the cell strength or targeting the cellular activation of photoreceptors in pathologic retinas, especially guiding the M opsin protein localization, should be involved. Nonetheless, the cellular mechanism by which crocin modulates phototransduction requires further study.

However, the clinical efficacy of crocin in functional vision rescue might be dependent on the degeneration, residual cellular conditions, and residual photoreceptor plasticity of the retina. Moreover, the purity of crocin or crocetin content in the supplement extract, as well as the dosage or duration of use, might also affect its therapeutic efficacy in clinical settings. As a result of the current clinical trials, oral administration of crocin (15 mg/daily) for 3 months could significantly improve BCVA in patients with refractory diabetic maculopathy [16], whereas crocin-rich saffron (20 mg/daily) treatment for 90 days could significantly improve retinal flicker sensitivity and BCVA in patients with mild/moderate age-related macular degeneration (AMD) [15,17]. In contrast, crocin-rich saffron (20 mg/daily) for over a 6-month period did not show a significant change in fERG and VA in patients with ABCA4-related Stargardt macular dystrophy [29]. Nonetheless, crocin-rich supplementation is generally considered to be well-tolerated in humans, thereby producing greater benefits in vision healthcare in patients with retinal disease. However, this will have to be proven through further research.

In summary, our results demonstrate the functional vision restoration effects of crocin on cone photoreceptors in a mouse model of LED bright light-induced retinal degeneration. The rescuing or restoring effects of crocin on the threshold values of the VA and VCSF are primarily reflected in the high spatial-frequency-based visual performance, which involves the activation of the BDNF–TrkB pathway. In conclusion, crocin-rich ingredients could be used in eye healthcare for enhancing functional vision and protecting retinal tissues.

## Data Availability

Data available only on request due to ethical restrictions.
